# Modelling the cost-effectiveness of catch-up ‘MenB’ (Bexsero) vaccination in England

**DOI:** 10.1016/j.vaccine.2016.11.076

**Published:** 2017-01-05

**Authors:** Hannah Christensen, Caroline L. Trotter

**Affiliations:** a School of Social and Community Medicine, University of Bristol, Oakfield House, Oakfield Grove, Bristol BS8 2BN, England, United Kingdom; b Disease Dynamics Unit, Department of Veterinary Medicine, University of Cambridge, Madingley Road, Cambridge CB3 0ES, England, United Kingdom

**Keywords:** Meningococcal disease, Group B, Vaccination, Catch-up, Cost-effectiveness

## Abstract

We assessed the cost-effectiveness of offering catch-up vaccination with Bexsero against meningococcal disease to children too old to receive the vaccine under the recently introduced infant programme. Offering catch-up vaccination to increasingly older children is less economically attractive because of declining disease burden. We estimate catch-up vaccination of 1 year old children could be cost-effective, incremental on the infant programme with a vaccine price of ⩽£8 per dose. Extending vaccination to 2 year olds could only be cost-effective (incremental on infant and 1 year old catch-up) with a vaccine price of ⩽£3 per dose and was not cost-effective in sensitivity analyses with more conservative vaccine assumptions. Extending catch-up further to 3–4 year olds was not cost-effective. Employing the current criteria for assessing vaccines, our models suggest that even with low vaccine prices only catch-up vaccination in 1 year old children could be cost-effective, when considered incrementally on the infant programme.

## Highlights

• We modelled the cost-effectiveness catch-up ‘MenB’ vaccination in England.
• Catch-up vaccination for 1 year old children could be cost-effective at ⩽£8 per dose.
• Additionally vaccinating 2 year olds was less cost-effective.
• With conservative vaccine assumptions vaccinating 2 year olds was not-cost-effective.
• It was not cost-effective to extend vaccination further to 3–4 year olds.

## 1 Introduction

In September 2015 the UK became the first country in the world to routinely offer to infants a vaccine (Bexsero) against MenB disease at 2, 4 and 12 months of age. The decision to immunise infants was made by the Joint Committee on Vaccination and Immunisation (JCVI), based on evidence that this age group are the most at risk of invasive disease and that immunising this group could be cost-effective if the vaccine was procured at a low price [1].

In spring 2016 the largest health petition in UK history was received by parliament, calling for vaccination against meningococcal group B disease (MenB) for all children up the age of 11 years [2]. As part of the original vaccine decision making process, there were several iterations of mathematical and economic models, which considered many different vaccination strategies, including catch-up strategies targeting pre-school (1–4 years) or school-aged (5–17 years) children [3], [4]. One of the key uncertainties is whether Bexsero can prevent transmission of the meningococcus and induce herd protection [5]. Assuming that the vaccine provides direct protection only, our previous models have shown that catch-up vaccination was unlikely to be cost-effective, and therefore could not be recommended by JCVI.

The aim of this modelling study was to further investigate the cost-effectiveness of different catch-up options, focusing not on children under 11 years, but on the birth cohorts after infancy who experience the greatest disease burden, i.e. 1, 2 and 3–4 year olds.

## 2 Methods

We adapted the transmission dynamic mathematical and economic model used to inform the infant vaccination JCVI decision [3] to consider additional catch-up vaccination options. We modelled vaccination as a two dose schedule delivered 1 month apart in catch-up cohorts (vaccine uptake in catch-up cohorts was assumed to be the same as for the MenC vaccine campaign [6]). In the base case we assumed the vaccine covered 88% [7] of circulating meningococcal strains with a 30% vaccine efficacy against carriage acquisition [5] and 95% vaccine efficacy against disease [8], [9].

We used the same parameter values in the model as considered previously [3], except for the price for the vaccine delivery cost. The fee given to GPs for administering vaccines and immunisations increased from £7.50 to £9.80 per dose from 1 April 2016 [10]. The model includes costs of: acute hospital care and initial follow-up appointments, public health response, long term support for survivors with sequelae, and litigation claims against the NHS. Disease incidence and case fatality estimates were drawn from Hospital Episode Statistics data over a seven year (2005/06–2011/12) period to allow for potential future increases in disease; in a historical context the UK is currently experiencing low disease incidence. Vaccine delivery costs are modelled separately from the cost of the vaccine and costs of adverse reactions are also considered. The benefits of vaccination are captured through gains in Quality Adjusted Life Years (QALY) through reducing disease cases, sequelae and death; QALY gains in family and network members were considered in sensitivity analyses. Previously the JCVI specified a QALY adjustment factor of x3 should be applied in the model for long-term sequelae due to concerns surrounding the ability of current tools to adequately capture quality of life losses due to meningococcal disease. This adjustment factor was retained in this iteration of the model.

The cost-effectiveness of catch-up vaccination was considered incrementally on the existing routine infant programme; we did not consider catch-up vaccination beyond 4 year olds because our previous work (data not shown [3]) had indicated this would not be cost-effective. Future costs and benefits were discounted at 3.5% in the base case and analyses were undertaken from the NHS and personal and social services perspective. Strategies were deemed cost-effective if the discounted cost per QALY gained was <£20,000 for the base case. For consistency with the routine infant immunisation decision by JCVI we considered a conservative scenario assuming 66% strain coverage for the vaccine and no herd effects (no vaccine efficacy against carriage acquisition); this was deemed cost-effective if the discounted cost per QALY gained was <£30,000 [11], [12]. We also assessed the effect of using 1.5% discounting for cost and benefits, including family and network QALYs, assuming a lower incidence and returning to the previous vaccine delivery cost of £7.50 per dose.

## 3 Results

In the base case model, catch-up vaccination of 1 year old children (83.8% uptake) with Bexsero could be considered cost-effective, incremental on the existent routine infant programme, if the vaccine could be procured at a low vaccine price, estimated at ⩽£8 per dose with a threshold of £20,000 per QALY gained (Table 1). Extending the catch-up to include 2 year olds (75.6% uptake) was less economically attractive, driven by the fact that disease incidence in 2 year olds is lower than in 1 year old children; in the absence of vaccination the model assumes 359 cases in infants (annual incidence 52.9/100,000 persons, 0 year olds), 193 cases in 1 year olds (28.5/100,000) and 111 cases in 2 year olds (16.4/100,000). We estimated that catch-up vaccination in 2 year olds, incremental on the routine infant programme and 1 year old catch-up, could only be cost-effective if the vaccine were priced £3 per dose or less. It was not possible to find a positive vaccine price when extending catch-up further to 3 and 4 year olds (annual incidence 11.2 and 8.4/100,000 respectively; 75.6% vaccine uptake in both year groups), and since disease incidence falls further after this age, the same applies up to age 11 years.

Reducing the discounting for costs and benefits from 3.5% to 1.5% improves the incremental cost-effectiveness ratio and increases the threshold vaccine price. In this scenario catch-up vaccination in 1 year olds could be cost-effective at £20 per dose, incremental on the infant programme, extending this to 2 year olds and then to 3–4 year olds could be incrementally cost-effective at £12 and £6 per dose respectively.

If the previous vaccine delivery cost of £7.50 per dose is used instead of the new £9.80 fee, the estimated ‘cost-effective’ vaccine prices for 1 and 2 year old catch-up are increased by £2.30 a dose and extending catch-up to 3–4 year olds could then be deemed cost-effective at a vaccine price ⩽£2 per dose.

Disease incidence naturally varies over time even in the absence of intervention against MenB disease. The base case model uses incidence over a seven year period to allow for such future changes in disease, but this is higher than the low burden currently experienced. Reducing the modelled number of annual cases of disease by a third, to more closely resemble the incidence experienced currently, rules out many catch-up strategies from an economic perspective. Only 1 year old catch-up could be cost-effective in this scenario and only with a vaccine price of ⩽£1 per dose.

In the conservative scenario with 66% vaccine strain coverage and no herd effects (all other parameters at base case values), using a threshold of £30,000 per QALY gained, only catch-up vaccination in 1 year old children could be considered cost-effective and only with a very low vaccine price of ⩽£2 per dose (Table 2). However, if family and network QALYs were included this could be increased to £6 per dose. Under extremely conservative assumptions (lower disease incidence, 66% strain coverage, no herd effects or litigation costs) none of the catch-up policies could be considered cost-effective with 3.5% discounting. Conversely using highly favourable assumptions (91% strain coverage, 60% vaccine efficacy against carriage, including litigation costs and quality of life losses in family and network members, with 1.5% discounting) catch-up vaccination of 1, 2 and 3–4 year olds could be incrementally cost-effective at £35, £24 and £18 per dose respectively with a willingness to pay of £20,000 per QALY gained.

## 4 Discussion

Our model estimates that offering Bexsero to 1 year old children in a catch-up campaign could be cost-effective at £8 per dose with a willingness to pay of £20,000 per QALY. Providing catch-up vaccination to older birth cohorts is less economically attractive due to a decreasing disease burden, and even extending vaccine to 2 year olds could not be considered cost-effective.

A strength of this work is that it builds upon a previously independently reviewed model which was used to inform the infant recommendation for the use of Bexsero in the UK [11]. There remains, however a considerable degree of uncertainty around many of the model parameters and whilst surveillance data will help to reduce some of this uncertainty, it is still too early in the programme to be able to revise any of the assumptions, for example of vaccine effectiveness. To our knowledge this is the first study to consider the cost-effectiveness of extending catch-up vaccination in individual birth cohorts after infancy. Previous models have either considered routine programmes only [13], [14] or catch-up in a number of birth cohorts combined [3], [15], [16].

Although our models suggest catch-up vaccination in 1 year olds could be cost-effective, it may be challenging to achieve this in practice. Our base case models assume the vaccine has some ability to disrupt transmission and carriage although the evidence for this is limited at present [5]. It is thought that carriage prevalence is low in young children [17], thus the potential herd effects generated through vaccinating this age group may not be large. However, excluding any effect on carriage reduces the already low cost-effective vaccine price. The price procured per dose of vaccine for the infant programme is confidential. Thus while the price per dose for 1 year old catch-up appears similar to that estimated for the infant programme, if additional factors had to be included for a vaccine price to be agreed for the infant programme (such as the removal of an infant meningococcal C conjugate vaccine dose from the current schedule [11]), procurement of doses for catch-up may not be possible at the prices we have suggested. Changes have also recently been made to the vaccine delivery cost and while there is currently no scope for reverting to the previous cost, our findings highlight that vaccine recommendation decisions can be affected by the administration fee. The window of opportunity to vaccinate these individuals is also limited. Babies born after 1 May 2015 are already eligible for vaccination through the NHS infant schedule, thus the cohort of toddlers who are aged 1 and who have not been vaccinated is reducing. Given the seasonal nature of meningococcal disease any catch-up vaccination would need to be done sufficiently early to afford protection to these children before the winter peak in disease to maximise the benefit from immunisation.

## 5 Conclusions

Based on the criteria currently used by JCVI our models suggest only catch-up vaccination in 1 year old children could be recommended on economic grounds, incremental to the existing infant programme, but only if the vaccine could be procured at a low cost.

## Funding

The research was funded by the National Institute for Health Research Health Protection Research Unit (NIHR HPRU) in Evaluation of Interventions at the University of Bristol in partnership with Public Health England (PHE). The views expressed are those of the author(s) and not necessarily those of the NHS, the NIHR, the Department of Health or Public Health England. The sponsors of the study had no role in study design, data collection, data analysis, data interpretation, or writing of the report. HC will act as guarantor.

## Conflict of interest

CLT reports receiving a consulting payment from GSK in 2013 and an honorarium from Sanofi Pasteur in 2015. HC reports receiving an honorarium from Sanofi Pasteur in 2015 and 2016, and consultancy fees from IMS Health and AstraZeneca all paid to her employer.

## Figures and Tables

**Table 1:** Cost-effectiveness of catch-up vaccination assuming 88% vaccine strain coverage, 30% vaccine efficacy against carriage acquisition and 95% vaccine efficacy against disease.

Vaccine strategy	Vaccination strategy compared with no vaccination	Catch-up vaccination incremental on previous (row above) strategy^a^		
	ICER at £75/vaccine dose^b^	ICER at £75/vaccine dose^b^	Vaccine price at £30 k/QALY^c^	Vaccine price at £20 k/QALY^c^
*3.5% discounting for costs and benefits*				
2,4 + 12 months	£168,000	–	–	–
2,4 + 12 months + CU in 1 y	£167,400	£143,200	£13	£8
2,4 + 12 months + CU in 1–2 y	£167,900	£199,800	£7	£3
2,4 + 12 months + CU in 1–4 y	£170,100	£264,800	£2	NP

*1.5% discounting for costs and benefits*				
2,4 + 12 months	£114,200	–	–	–
2,4 + 12 months + CU in 1 y	£113,600	£79,000	£29	£20
2,4 + 12 months + CU in 1–2 y	£113,600	£111,000	£19	£12
2,4 + 12 months + CU in 1–4 y	£114,100	£147,800	£12	£6

ICER, incremental cost-effectiveness ratio; QALY, Quality Adjusted Life Year; CU, catch-up vaccination; NP, positive vaccine price not possible.

a 2,4 + 12 months + CU in 1 y incremental on 2,4 + 12 months; 2,4 + 12 months + CU in 1–2 y incremental on 2,4 + 12 months + CU in 1 y etc.

b Figures rounded to nearest 100.

c Figures rounded to nearest £1.

**Table 2:** Cost-effectiveness of catch-up vaccination assuming 66% vaccine strain coverage, 0% vaccine efficacy against carriage acquisition and 95% vaccine efficacy against disease.

Vaccine strategy	Vaccination strategy compared with no vaccination	Catch-up vaccination incremental on previous (row above) strategy^a^		
	ICER at £75/vaccine dose^b^	ICER at £75/vaccine dose^b^	Vaccine price at £30 k/QALY^c^	Vaccine price at £20 k/QALY^c^
*3.5% discounting for costs and benefits*				
2,4 + 12 months	£273,400	–	–	–
2,4 + 12 months + CU in 1 y	£273,100	£262,700	£2	NP
2,4 + 12 months + CU in 1–2 y	£274,800	£401,800	NP	NP
2,4 + 12 months + CU in 1–4 y	£280,300	£613,700	NP	NP

*1.5% discounting for costs and benefits*				
2,4 + 12 months	£188,600	–	–	–
2,4 + 12 months + CU in 1 y	£188,100	£151,100	£11	£6
2,4 + 12 months + CU in 1–2 y	£188,500	£233,000	£4	NP
2,4 + 12 months + CU in 1–4 y	£190,300	£358,300	NP	NP

ICER, incremental cost-effectiveness ratio; QALY, Quality Adjusted Life Year; CU, catch-up vaccination; NP, positive vaccine price not possible.

a 2,4 + 12 months + CU in 1 y incremental on 2,4 + 12 months; 2,4 + 12 months + CU in 1–2 y incremental on 2,4 + 12 months + CU in 1 y etc.

b Figures rounded to nearest 100.

c Figures rounded to nearest £1.
